# Obsessive Compulsive Symptoms, Obsessive Compulsive Disorder and Related Disorders in Idiopathic Parkinson’s Disease

**DOI:** 10.1192/j.eurpsy.2023.525

**Published:** 2023-07-19

**Authors:** E. Çakıroğlu, E. Ozan, M. Batum

**Affiliations:** ^1^Psychiatry; ^2^Neurology, Manisa Celal Bayar University Faculty of Medicine, Manisa, Türkiye

## Abstract

**Introduction:**

Obsessive-compulsive symptoms and Obsessive-Compulsive Disorder (OCD) have been reported by clinicians in Parkinson’s disease (PD) due to the involvement of a shared circuitry. In DSM-5, OCD related disorders include Trichotillomania, Hoarding Disorder, Skin Picking Disorder, and Body Dysmorphic Disorder. However, there is no prevalence information about Obsessive Compulsive and Related Disorders in PD.

**Objectives:**

The aim of this study is to evaluate patients with Idiopathic Parkinson’s Disease (IPD) in terms of obsessive-compulsive symptoms, OCD and Related Disorders, compare with a matched control group, and determine the relationship between the two phenomena.

**Methods:**

80 patients between 52-82 years of age, diagnoised IPD and 80 healthy controls between 52-80 years of age were included in this study. Structured Clinical Interview for DSM-5 Disorders, a Structured sociodemographic and clinical form, The Unified Parkinson’s Disease Rating Scale, Frontal Assessment Battery (FAB), Parkinson’s Disease Questionnaire-39 (PDQ-39) and Yale-Brown Obsessive-Compulsive Scale were applied to the participants. The relationship between PD and OCD and related disorders and obsessive-compulsive symptoms was evaluated.

**Results:**

Contamination obsession was found in 21.3% of patients and 16.3% of the control group; cleaning compulsion was found in 13.8% of patients and 8.8% of the control group. Two groups were compared in terms of obsessions (p=0.215) and compulsions (p=0.361); no statistically significant difference was found between the groups.The presence of obsessions (p=0.027) and compulsions (p=0.007) in patients with a family history of Parkinson’s disease was found to be significantly higher. A statistically significant correlation was found between FAB scores and PDQ-6 (cognition item) in the Parkinson’s group (r= -0.359, p=0.001). Compared to the control group (mean 15,08; SD: 1,84), FAB scores were found to be significantly lower in the Parkinson’s group (mean 13,95; SD: 2,23) .(p=0.001). The presence of any obsession (p=0.003) or any compulsion (p<0.001) was found to be significantly higher in patients with hoarding in the Parkinson’s group. The presence of any obsession (p<0.001) or any compulsion (p<0.001) was found to be significantly higher in patients with Skin Picking Disorder in the Parkinson group. The scores obtained from the PDQ emotional well-being (p=0.027) and bodily discomfort (p=0.001) items in the Parkinson group with Skin Picking Disorder were significantly higher than the Parkinson group without Skin Picking Disorder.

**Conclusions:**

PD is characterized by dysfunction in frontobasal ganglia circuits. A similar circuit is also important in OCD. Obsessions and compulsions, Hoarding Disorder, Skin Picking and Trichotillomania may accompany PD. Family history increases any obsession and compulsion in PD.

**Disclosure of Interest:**

None Declared

